# Optimizing Cancer Survivorship Care: Examination of Factors Associated with Transition to Primary Care

**DOI:** 10.3390/curroncol30030207

**Published:** 2023-02-24

**Authors:** Som. D. Mukherjee, Daryl Bainbridge, Christopher Hillis, Jonathan Sussman

**Affiliations:** Juravinski Cancer Center, Hamilton Health Sciences, Department of Oncology, McMaster University, Hamilton, ON L8S 4L8, Canada

**Keywords:** transitions in care, primary health care, cancer survivorship, risk stratification

## Abstract

Healthcare systems in Canada and elsewhere have identified the need to develop methods to effectively and safely transition appropriate cancer survivors to primary care. It is generally accepted that survivors with a low risk of adverse events, including recurrence and toxicity, should be more systematically identified and offered transition. There remains a lack of clarity about what constitutes an appropriate profile that would assist greater application in practice. To address this gap, we examined the clinical profiles of patients that were transitioned from a large regional cancer centre to the community. The factors examined included disease site, clinical stage, time since diagnosis/first consult, cancer treatments, and Edmonton Symptom Assessment System (ESAS) scores. In total, 2604 patients were identified as transitioned between 2013 and 2020. These patients tended to have common cancers (e.g., breast, endometrium, colorectal) that were generally of lower stage. Half of the patients had received chemotherapy and/or radiation treatment. Nearly one-third of survivors were transitioned within a year of first consult and a third after five years. Most patients reported minimal symptoms based on ESAS scores prior to being transitioned. This study represents one of the first to analyze the types of cancer patients that are being selected for transition to primary care.

## 1. Introduction

Advances in cancer treatment over the past few decades have led to improvements in cancer survival [1,2,3]. As the number of cancer patients surviving their illness increases over time, there is a growing need in healthcare to re-evaluate how to provide care for these patients. Survivorship care often includes surveillance for cancer recurrence as well as ongoing management of early and late effects from both the cancer and cancer treatment [4] Cancer centres are increasingly challenged with limited resources to provide optimal care for cancer patients and survivors [5,6,7,8].

Several randomized controlled trials have provided evidence to support the practice of transitioning cancer patients from a cancer centre to their primary care practitioner for ongoing cancer care (e.g., survivorship care), following the completion of their primary cancer treatments [9,10,11,12]. These studies demonstrated that patients generally receive a high quality of care, regardless of whether they are followed at a cancer centre by their oncologist or by their primary care provider. Patient satisfaction and quality of life were also shown to be similar, regardless of where cancer patients are followed, although studies have demonstrated that some patients can be anxious about having their care transitioned away from the cancer centre [13,14,15]. 

There is wide variability among oncologists with respect to how they select patients for transition from the cancer centre to the community [16,17]. While it is generally assumed that survivors with lower risk of recurrence and adverse events are more likely to be transitioned, there is a paucity of research evaluating the manner with which clinicians select patients for transition of their cancer follow-up care to their primary care practitioner, and which patient or cancer-specific factors are used to identify these patients. Data on the types of patients that cancer care providers transition to primary care can help inform interventions towards a more systematic approach to choosing this care pathway.

We were in a unique position at our institution to study these patients being transitioned from a cancer centre, as all discharged cancer patients have been designated a unique transition patient identification code since 2013. The primary aim of our study was to examine the demographic profiles and cancer-specific characteristics from a large cohort of oncology patients that had their oncology care transitioned from a cancer centre, to improve our understanding of how patients are being identified for transition by their oncologist. Our approach is based on the assumption that those judged to be at low risk of adverse outcomes (e.g., recurrence or side effects of treatment) are most likely being transitioned to primary care.

## 2. Methods

We completed a retrospective single cohort study of transitioned (cases) cancer patients using the MOSAIQ Electronic Medical Record (EMR) at the Juravinski Cancer Centre (JCC) in Hamilton, Ontario. The JCC is a large regional cancer centre serving approximately 23,000 patients annually. The EMR at the JCC has a system which allows clinical staff to use a Transition Code (T-Code) to indicate when a patient is being transitioned to their primary care provider. A study database was created by the JCC Health Informatics Team for all cancer patients at the JCC with a T-Code. Patient and clinical factors from the EMR were linked to this database. An exploratory analysis of factors potentially prognostic for transition was completed using summary descriptive statistics (means for continuous variables and proportions for categorical variables). Patient-reported symptoms, as measured by the Edmonton Symptom Assessment System (ESAS) scale, were also recorded at our centre starting in 2015. The ESAS is a validated tool designed to screen for self-reporting the severity of nine different symptoms on a scale of 0–10 (10 = worst), including anxiety, appetite, depression, drowsiness, nausea, pain, shortness of breath, tiredness, and well-being [18]. ESAS scores between 4 and 6 are considered moderate in severity for a given symptom, while scores between 7 and 10 are considered severe [19].

Measures were taken, including crosschecking with patients’ charts, to ensure the veracity and completeness of data. We ascertained the validity of the T-Code designation in the record by confirmation with the notes of the responsible oncologist. Data extraction for all patients in the study included a comprehensive summary of baseline clinical and demographic information, tumour disease type and stage at diagnosis, details of prior chemotherapy (defined as any systemic treatment given in the chemotherapy suite), radiation and surgical (if available) treatment, date of transition and patient-reported outcomes (ESAS). Ethical approval for this study was obtained from the Hamilton Integrated Research Ethics Board (#8090).

## 3. Results

### 3.1. Transitioned Cohort

In total, 2604 patients were identified with a T-Code and a confirmed diagnosis of cancer, indicating that the survivorship care of these patients was transferred to primary care. Most data variables were complete, except for TNM stage group information (Tumour, Nodes, Metastases) and ESAS scores in the month prior to transition. A manual chart abstraction was completed to obtain missing stage data. 

The T-Codes reported included transitioned cancer patients between 30 September 2013 and 27 February 2020. The number of patients recorded as having transitioned per year increased from 169 in 2014 when the study timeframe began to 370 in 2019 when data collection ended. To verify the reliability of the T-Code data, the study investigators (SM, JS, and DB) reviewed a 5 percent random sample (130 cases) of the total cohort and found the transition to be confirmed in over 99% (129 of 130) of cases.

### 3.2. Demographic Factors

The majority (80% (2086)) of transitioned patients were female. The median age of patients transitioned was 66 (min 20, max 96, standard deviation (SD) = 11.8). In total, 9% of patients in our study were between the ages of 18 and 49, 21% between ages 50 and 59, 32% between ages 60 and 69, and 38% of patients were 70 years or older. 

Patients with breast cancer represented the most common tumour site in our cohort (47%), followed by endometrial cancer (20%), colorectal cancer (8%), prostate cancer (6%), and melanoma (5%) (Table 1). The remaining cancer sites were much less frequent and evenly distributed between female and male patients.

### 3.3. Cancer Stage

We used the most appropriate clinical staging group by disease site at the time of transition (Table 1). The majority of transitioned patients had early-stage cancer (stage 0 to 2), with 50% of patients being Stage 0 or 1 and 31% being Stage 2 (Table 1). This finding was consistent across nearly all cancer disease sites. One exception was lower gastrointestinal cancers, the majority (111, 59%) of which were Stage 3 at transition. This finding is reflective of the fact that most early-stage colorectal cancer patients do not receive adjuvant therapies after surgery and are less frequently seen in consultation at a cancer clinic. Some patients identified as Stage 4 (4.7%) were transitioned, likely after having discontinued active treatment.

### 3.4. Time from Initial Consultation to Transition

**Figure 1:**
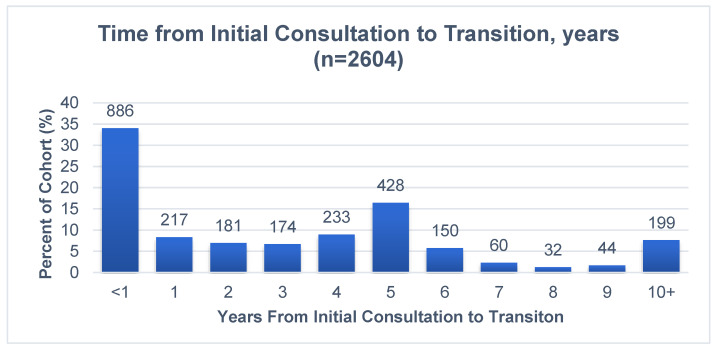
Time from initial consultation to transition, years.

On average, the transitions occurred 3.9 years after the initial consultation at the JCC (median 3.1 years, SD 4.5). Distribution of time to transition demonstrated peaks at within one year and in the fifth year, with approximately one-third (34%) of patients transitioned within a year of the initial consult, while a similar proportion (35%) was transitioned after five years (see Figure 1). About 11% of transitions took place between 6 and 10 years after initial consultation.

### 3.5. Cancer Treatment

Just over half (51%) of patients transitioned in our cohort had received prior chemotherapy and/or radiation treatment (Table 2). We were not able to reliably collect data on surgery or use of endocrine therapy from the cancer centre electronic medical records. Of patients who received no chemotherapy and/or radiation treatment, 45% (584) of these patients were transitioned within a year of initial consultation, while 36% (461) were transitioned after five years of follow-up. Melanoma, as well as endometrial, hematological, and non-melanoma skin cancers were most represented among those patients who had not received chemotherapy or radiation treatment.

### 3.6. Patient-Reported Outcomes

In the study cohort, 1187 patients had an ESAS score recorded within 1 month of transition from the cancer centre. Most of these patients (73% to 98%) had ESAS scores of less than 4 for a given symptom (see Table 3).

## 4. Interpretation

This retrospective analysis of cancer patients transitioned from a cancer centre provides a unique and more detailed profile of the types of patients that community-based providers are likely to see in survivor follow-up. Overall, oncologists most commonly transitioned patients with a primary diagnosis of breast, endometrial, colorectal, or prostate cancer. These findings are not unexpected as these tumour disease sites represent the most common malignancies encountered in clinical oncology practice. In addition, patients with lower-stage tumours (i.e., Stage 1 or 2) that would typically be associated with a lower risk of recurrence made up a large proportion of the transitioned cohort (76%), as compared to patients with more advanced Stage 3 or 4 tumours (18%). We observed that a large proportion of transitions took place within the first year after consultation. Further analysis of this subgroup demonstrated that nearly two-thirds of these patients had not received treatment with either chemotherapy or radiation. Of the transitioned cohort who had received chemotherapy or radiation, we observed a wide range of diagnoses, stages, and treatment approaches (type and duration of chemotherapy and radiation therapies).

A review of the literature reveals one other recent study that reported the detailed characteristics of transitioned cancer patients. Nguyen et al. examined the characteristics of 148 thyroid cancer patients transitioned from oncology care to a primary-care-based follow-up clinic [20]. The study found that the transitioned patients had surgical treatment, 68% total thyroidectomy, the mean age of these patients was 55 years, 16% had other cancer diagnoses, nearly all were Stage 1 (83%) or Stage 2 (13%), and the mean time between diagnosis and transition was 4.5 years. This study suggested the approach was clinically reasonable for early-stage patients given that the transitioned patients (since program inception in 2010) showed no evidence of recurrent disease and did not appear to require re-referral to oncology. In contrast to our approach, no patient-reported outcomes were reported in this study.

There are few guidelines available to support oncologists in identifying cancer patients for transition but, to date, the criteria for this decision are largely left to the option and discretion of the individual clinician. Cancer Care Ontario published practice guidelines on transitioning cancer survivors to their family physician that recommend this practice, but without any specificity on how oncologists should identify these patients [21,22,23]. The National Health Service (NHS) published and implemented risk-stratified cancer survivorship guidelines in the United Kingdom for patients with breast, colorectal, and prostate cancers [24]. The NHS strategy emphasises post-treatment transition from specialist care, with the caveat that this pathway choice is ultimately a joint decision between the cancer survivor and their oncologist. This decision depends on a number of factors, including: level of risk associated with cancer type; short- and long-term effects of treatment; other co-morbidities; patient’s ability to manage; and level of professional involvement required. The guideline encourages clinicians to develop and test their own specific criteria for risk stratification/transition to primary care. While the principle of risk-stratified pathways for cancer survivorship is endorsed in many healthcare systems [8,25], there remains a lack of detail on how to apply these general guidelines into practice [26]. Further, more in-depth research evaluating clinical factors to consider when selecting patients would be valuable, including variables, such as general risk of cancer recurrence based on specific tumour pathology characteristics and symptoms patients may be experiencing, including emotional distress.

Our analysis included examination of patient-reported outcomes using universally collected ESAS scores. Many cancer patients are known to suffer from long-term side effects and emotional distress in the months and years following diagnosis [27]. Our study found that patients transitioned from the cancer centre had generally low self-reported symptom scores (as measured by ESAS screening data) prior to transition that may suggest oncologists are considering symptoms when deciding on which patients to identify for transition. Further research evaluating post-transition patterns of care for survivors that include returning to the cancer centre for subsequent care would also be of interest towards understanding clinical outcomes for transitioned survivors.

## 5. Limitations

As a single cohort study, there are several limitations to acknowledge that limit the robustness of our observations. Our study did not include a comparison group, which limits the ability to determine if the profiles we observed are truly unique to the transitioned survivor population. We were also unable to collect reliable data on how many patients underwent surgical procedures and the type of procedure due to many patients having surgery at institutions outside of our cancer centre. We were also not able to reliably assess endocrine therapy use for breast and prostate cancer patients as many patients filled their prescriptions at pharmacies outside the cancer centre. Our database did not include information on patient comorbidities so we were not able to review whether this may have affected patient selection for transition. Finally, many patients in our study cohort did not have an ESAS score around the time of transition. These missing ESAS data are due to them not being routinely collected during the initial years of the transition cohort database, but also patients not completing the ESAS at their last visit to the cancer centre.

## 6. Conclusions

We have been able to provide a relatively detailed snapshot of cancer survivors who have been transitioned from a large regional cancer centre to community providers. Survivors in this study had a variety of common types of cancer, which were generally of lower stage, associated with a lower risk of recurrence. In addition, patients tended to have minimal symptoms based on ESAS scores prior to being discharged to the community. This represents the first study of its kind to analyze the detailed profiles of cancer patients that are being transitioned to the community. Our findings contribute to the understanding of how survivors may be considered for transition and suggest that a “low risk” profile could be systematically identified from existing cancer centre EMR data.

Within a relatively large pool of oncology patients seen at our institution over several years, we found that the specific use of transition codes in this study helped us to systematically identify the small group of cancer patients transitioned to their primary care practitioner for research analyses. Researchers involved in transitions research may wish to consider using transition codes as part of their study methodology as a simple yet effective tool to identify such patients. In addition, we believe that collaborative research partnerships, such as those that have been described in pediatric cancer cohorts [28] that use a common methodology, would support much larger study cohorts of transitioned adult cancer survivors and a better understanding of how these patients are identified and managed in daily clinical practice.

We plan future research to confirm and enhance the robustness of the findings of this study and to ultimately inform the systematic identification of cancer patients who are most appropriate for safe and effective transitions.

## Figures and Tables

**Table 1:** Clinical stage at the time of transition, n = 2604.

Diagnosis		Clinical Stage							Total
		0	1	2	3	4	No Stage Recorded	Non-Malignant Condition	
Breast	N	123	497	472	110	11	4	0	1217
	%	10.1	40.8	38.8	9.0	0.9	0.3	0.0	46.7
Endometrium	N	4	448	33	18	9	0	0	512
	%	0.8	87.5	6.4	3.5	1.8	0.0	0.0	19.7
Gastrointestinal-lower	N	0	16	64	122	7	0	0	209
	%	0.0	7.7	30.6	58.4	3.3	0.0	0.0	8.0
Prostate	N	1	44	99	20	5	0	0	169
	%	0.6	26.0	58.6	11.8	3.0	0.0	0.0	6.5
Melanoma and skin	N	0	62	34	19	3	0	0	118
	%	0.0	52.5	28.8	16.1	2.5	0.0	0.0	4.5
Hematological	N	1	29	26	11	21	8	6	102
	%	1.0	28.4	25.5	10.8	20.6	7.8	5.9	3.9
Genitourinary *	N	0	19	33	7	8	1	0	68
	%	0.0	27.9	48.5	10.3	11.8	1.5	0.0	2.6
Gynecological **	N	5	28	8	11	5	0	2	59
	%	8.5	47.5	13.6	18.6	8.5	0.0	3.4	2.3
Gastrointestinal-upper	N	0	5	13	6	24	1	0	49
	%	0.0	10.2	26.5	12.2	49.0	2.0	0.0	1.9
Sarcoma	N	7	10	15	6	4	0	2	44
	%	15.9	22.7	34.1	13.6	9.1	0.0	4.5	1.7
Head and neck	N	1	5	9	5	12	0	0	32
	%	3.1	15.6	28.1	15.6	37.5	0.0	0.0	1.2
Lung	N	0	2	3	5	6	0	0	16
	%	0.0	12.5	18.8	31.3	37.5	0.0	0.0	0.6
Central nervous system	N	0	0	0	0	3	0	0	3
	%	0.0	0.0	0.0	0.0	100	0.0	0.0	0.1
other Malignant Neoplasm	N	0	0	0	0	1	0	0	1
	%	0.0	0.0	0.0	0.0	100	0.0	0.0	0.0
Unknown Malignant Neoplasm	N	0	0	1	0	4	0	0	5
	%	0.0	0.0	20.0	0.0	80.0	0.0	0.0	0.2
Total	N	142	1165	810	340	123	14	10	2604
	%	5.5	44.7	31.1	13.1	4.7	0.5	0.4	100.0

* inc cervix uteri, bladder, testis; ** inc vagina, vulva, ovary.

**Table 2:** Time to transition by treatment type, n = 2604.

	* Transition < 1 year (n = 886)		* Transition ≥ 1 year (n = 1718)		All Transitions	
Treatment **	N	% Total	N	% Total	N	% Total
Prior chemotherapy	199	7.6	744	28.6	943	36.2
Prior radiation	196	7.5	654	25.1	850	32.6
No chemotherapy or radiation received (tumour site, % of disease site total)						
Melanoma/skin (83.1%)	26	1.0	72	2.8	98	3.8
Hematological (81.4%)	16	0.6	67	2.6	83	3.2
Endometrium (77.9%)	344	13.2	55	2.1	399	15.3
Prostate (54.4%)	8	0.3	84	3.2	92	3.5
Gastrointestinal-upper (51.0%)	17	0.7	8	0.3	25	1.0
Gastrointestinal-lower (45.9%)	60	2.3	36	1.4	96	3.7
Breast (29.3%)	81	3.1	275	10.6	356	13.7
Other (58.8%)	32	1.2	106	4.1	138	5.3

* Time from initial visit to transition date. ** Note: Prior chemotherapy and radiation groups are not mutually exclusive; this includes 476 patients that received both chemotherapy and radiation treatment.

**Table 3:** ESAS domain score categorized and mean score (n = 1187).

ESAS Domain(s)	Less than 4		4 or Greater		Mean (SD)
	Count	%	Count	%	
Pain	1036	87.3%	151	12.7%	1.2 (2.1)
Tiredness	870	73.3%	317	26.7%	2.3 (2.5)
Drowsiness	1044	88.0%	143	12.0%	1.2 (2.0)
Nausea	1162	97.9%	25	2.1%	0.3 (1.0)
Lack of Appetite	1079	90.9%	108	9.1%	0.8 (1.9)
Shortness of Breath	1073	90.4%	114	9.6%	0.9 (1.8)
Depression	1040	87.6%	147	12.4%	1.2 (2.1)
Anxiety	970	81.7%	217	18.3%	1.6 (2.3)
Wellbeing	907	76.4%	280	23.6%	2.0 (2.4)

## Data Availability

The data presented in this study are available upon reasonable request from the corresponding author.
